# New horizons for building pyrenoid-based CO_2_-concentrating mechanisms in plants to improve yields

**DOI:** 10.1093/plphys/kiac373

**Published:** 2022-08-12

**Authors:** Liat Adler, Aranzazú Díaz-Ramos, Yuwei Mao, Krzysztof Robin Pukacz, Chenyi Fei, Alistair J McCormick

**Affiliations:** Institute of Molecular Plant Sciences, School of Biological Sciences, University of Edinburgh, Edinburgh EH9 3BF, UK; Institute of Molecular Plant Sciences, School of Biological Sciences, University of Edinburgh, Edinburgh EH9 3BF, UK; Institute of Molecular Plant Sciences, School of Biological Sciences, University of Edinburgh, Edinburgh EH9 3BF, UK; Institute of Molecular Plant Sciences, School of Biological Sciences, University of Edinburgh, Edinburgh EH9 3BF, UK; Lewis-Sigler Institute for Integrative Genomics, Princeton University, Princeton, New Jersey 08544, USA; Institute of Molecular Plant Sciences, School of Biological Sciences, University of Edinburgh, Edinburgh EH9 3BF, UK

## Abstract

Many photosynthetic species have evolved CO_2_-concentrating mechanisms (CCMs) to improve the efficiency of CO_2_ assimilation by Rubisco and reduce the negative impacts of photorespiration. However, the majority of plants (i.e. C3 plants) lack an active CCM. Thus, engineering a functional heterologous CCM into important C3 crops, such as rice (*Oryza sativa*) and wheat (*Triticum aestivum*), has become a key strategic ambition to enhance yield potential. Here, we review recent advances in our understanding of the pyrenoid-based CCM in the model green alga *Chlamydomonas reinhardtii* and engineering progress in C3 plants. We also discuss recent modeling work that has provided insights into the potential advantages of Rubisco condensation within the pyrenoid and the energetic costs of the Chlamydomonas CCM, which, together, will help to better guide future engineering approaches. Key findings include the potential benefits of Rubisco condensation for carboxylation efficiency and the need for a diffusional barrier around the pyrenoid matrix. We discuss a minimal set of components for the CCM to function and that active bicarbonate import into the chloroplast stroma may not be necessary for a functional pyrenoid-based CCM in planta. Thus, the roadmap for building a pyrenoid-based CCM into plant chloroplasts to enhance the efficiency of photosynthesis now appears clearer with new challenges and opportunities.

## Teaser

Research on pyrenoid formation has led to key advances toward engineering an algal CO_2_-concentrating mechanism into C3 land plants, and a recent model predicts an optimized pathway for future work.

## Introduction

Sustainable food security for the growing population is a pressing issue for global agriculture (Horton et al., 2021). Following the “Green Revolution” in the 1960s, advances in conventional breeding approaches have provided continual improvements in the yields of crops that have generally kept track with population growth (Evans and Lawson, 2020). However, opportunities for further improvements are becoming increasingly challenging due to the negative impacts of climate change, higher animal feed demands for meat and dairy from rising urban populations, and the limited usage of the genetic diversity of crop germplasms (Long et al., 2015; Dusenge et al., 2018; Varshney et al., 2020; Moore et al., 2021). Efforts to overcome these challenges have driven the development of novel biotechnological and synthetic biology-based engineering approaches that should help to bolster breeding efforts and yield requirements, and facilitate progress toward a more sustainable circular economy (Shih et al., 2016; Lenaerts et al., 2019; Wolter et al., 2019; Barros et al., 2021; Sukegawa et al., 2021; Yang et al. 2021). Yield increases over the past several decades have been driven largely by increases in harvest index (i.e. the amount of grain relative to plant biomass) and selection for improvements in leaf canopy architecture to increase the efficiency of light capture (Long, 2006). In contrast, photosynthetic capacity (i.e. the efficiency of CO_2_ to biomass conversion) is a key crop trait that has remained largely unchanged despite decades of intensive selection by conventional breeding (Long et al., 2019). Thus, photosynthetic capacity has become a major target for enhancement by molecular engineering strategies.

Over the past decade, plant biologists have used engineering approaches to improve aspects of both the light-dependent and light-independent reactions of photosynthesis with promising levels of success. Accelerating the relaxation of the photosystem II photoprotective response has resulted in a 15% increase in dry weight (DW) biomass in field-grown tobacco (*Nicotiana tabacum*), while enhancing photosynthetic electron transport had a beneficial effect on growth rates in Arabidopsis (*Arabidopsis thaliana*) and tobacco (Chida et al., 2007; Kromdijk et al., 2016; Simkin et al., 2017b; Yadav et al. 2018). Efforts to increase flux through the Calvin–Benson–Bassham (CBB) cycle have resulted in improved CO_2_ assimilation rates in tobacco (*N. tabacum*), wheat (*Triticum aestivum*), rice (*Oryza sativa*), and maize (*Zea mays*), and led to increased DW biomass yields ranging from 20% to 80% (Simkin et al., 2015, 2017a; Driever et al., 2017; Salesse-Smith et al., 2018; Yoon et al., 2020). Furthermore, introducing synthetic bypasses to suppress photorespiration has increased DW biomass by up to 40% in field-grown tobacco and 28% in rice (South et al., 2019; Wang et al., 2020). However, Wang et al. (2020) also reported a reduced seed setting rate in rice and highlighted the potential need to re-coordinate the source–sink relationship to optimize yields in future transgenic lines. Similarly, efforts to enhance photoprotective relaxation were not as successful in Arabidopsis as in tobacco (Garcia-Molina and Leister, 2020). Together, the latter studies indicate that engineering strategies need to be adjusted depending on the plant species and fine-tuned for optimal integration with their native metabolism. Nevertheless, recent successful efforts to stack enhancements for electron transport, photorespiration efficiency, and CBB cycle flux in Arabidopsis and field-grown tobacco suggest that complex and integrated synthetic biology engineering approaches to improve photosynthetic capacity are achievable (Simkin et al., 2017a; López-Calcagno et al., 2019, 2020).

One of the more complex, but higher reward, strategies for improving photosynthetic capacity in C3 plants is the introduction of a CO_2_-concentrating mechanism (CCM). CCMs locally elevate the concentration of CO_2_ around Rubisco, thus bringing Rubisco carboxylation closer to its maximum rate and outcompeting the Rubisco oxygenation reaction. Many photosynthetic species have evolved CCMs, including: the biochemical C4 and C2 CCMs exemplified in plants by a two-cell, Kranz-type architecture (see recent reviews by Ermakova et al., 2020; Lundgren, 2020); the biophysical CCMs found in cyanobacteria that encapsulate the primary carboxylase enzyme Rubisco in carboxysomes (e.g. cyanobacteria); and finally, the biophysical CCM in eukaryotic algae and several species of hornwort bryophytes (i.e. non-vascular plants) where the Rubisco pool is condensed into a matrix within a non-membrane-bound micro-compartment in the chloroplast called the pyrenoid (see recent reviews by Hennacy and Jonikas, 2020; Barrett et al., 2021; Borden and Savage, 2021). Previous models have predicted that introduction of a C4-type CCM into current C3 crop cultivars could result in theoretical biomass gains of ca. 30%, while biophysical CCMs might result in gains of up to 60% (Price et al., 2011; McGrath and Long, 2014; Yin and Struik, 2017; Long et al., 2019). In both cases, introducing CCMs may also lead to improved efficiencies in nitrogen use (e.g. due to a reduced requirement for a large Rubisco pool) and water use (e.g. from decreased stomatal conductance due to increased CO_2_ availability for Rubisco) in C3 plants.

Researchers working to introduce the biophysical CCMs from cyanobacteria and algae into plants have made good progress in engineering carboxysome and pyrenoid components, respectively (Long et al., 2018; Atkinson et al., 2020). Furthermore, many of the algal CCM components appear compatible with plants, at least in terms of appropriate localization (Atkinson et al., 2016). However, the introduction of an active bicarbonate (${\text{HCO}}_{3}^{-}$) uptake transporter on the chloroplast envelope to increase the concentration of inorganic carbon (Ci, i.e. CO_2_ and hydrated Ci, such as ${\text{HCO}}_{3}^{-}$) in the chloroplast has long been perceived as a key requirement for a functional biophysical CCM (Rae et al., 2017). To date, efforts to successfully introduce active ${\text{HCO}}_{3}^{-}$ transporters into the chloroplast envelope are still ongoing (Rottet et al., 2021). More recently though, the central importance of active ${\text{HCO}}_{3}^{-}$ transporters has been challenged. In this review, we focus on efforts to engineer into plants the pyrenoid-based biophysical CCM, which is best characterized in the green algae *Chlamydomonas reinhardtii* (hereafter Chlamydomonas). First, we describe our current understanding of the components and regulation of the CCM pathways in Chlamydomonas. We then discuss the development of a recent chloroplast-scale reaction–diffusion model for the biophysical CCM in Chlamydomonas (Fei et al., 2022), which provides insights into the operational requirements and costs of a pyrenoid-based CCM, and a guide for ongoing engineering efforts.

## A brief overview of the shortcomings of C3 photosynthesis

Most plants, including major staple crops like wheat and rice, use the C3 photosynthetic pathway. C3 plants rely on passive diffusion of ambient CO_2_ into the chloroplast where Rubisco catalyzes the reaction between ribulose 1,5-bisphosphate (RuBP) and CO_2_ to produce 3-phosphoglycerate (3PGA) for use in the CBB cycle. The anatomy of C3 leaves tends to maximize gaseous diffusion through intracellular air spaces and minimize liquid-phase diffusion resistances (i.e. mesophyll conductance) to the site of carboxylation in the chloroplast (Cousins et al., 2020; Gago et al., 2020). Although this architecture assists the passive diffusion of CO_2_ through the leaf, the average steady-state CO_2_ concentration in C3 chloroplasts under high light is approximately 180 ppm (6 µM), which is less than half of current ambient air CO_2_ levels (i.e. 420 ppm [14 µM]) (Bunce, 2005; Terashima et al., 2006; Ainsworth and Long, 2021). The Michaelis–Menten constant of Rubisco for CO_2_ (*K*_c_) in C3 plants ranges from 7 to 30 µM at 25°C (Orr et al., 2016). Thus, in C3 plants, Rubisco is never saturated with its CO_2_ substrate and does not perform at its maximum achievable carboxylation rate (i.e. *V*_cmax_). C3 plants typically compensate for low chloroplastic Ci levels by investing large amounts of resources into Rubisco (i.e. approximately 25% of soluble protein in leaves) (Galmés et al., 2014). However, a further challenge arises—Rubisco is an “error-prone” enzyme that can also interact with oxygen, which likewise can diffuse into the leaf. Oxygenation of RuBP is a counter-productive reaction that can occupy up to a third of the active sites of Rubisco in C3 plants (Tcherkez, 2016). Instead of 3PGA, Rubisco-catalyzed oxygenation produces 2-phosphoglycolate (2PG), which inhibits two enzymes in the CBB cycle (i.e. triose phosphate isomerase and sedoheptulose 1,7-bisphosphate phosphatase) and must be recycled back to 3PGA through the photorespiratory salvage pathway (Fernie and Bauwe, 2020). While 2PG metabolism may play a regulatory role in carbon and nitrogen metabolism (Flügel et al., 2017; Busch et al., 2018, 2020; Timm, 2020; Shi and Bloom, 2021), photorespiration is generally considered an energetically wasteful process that results in a partial loss of previously fixed CO_2_, and reduces the overall efficiency of photosynthesis and subsequently crop yield potential (Zhu et al., 2010).

## Pyrenoid-based CCMs

**Figure 1:**
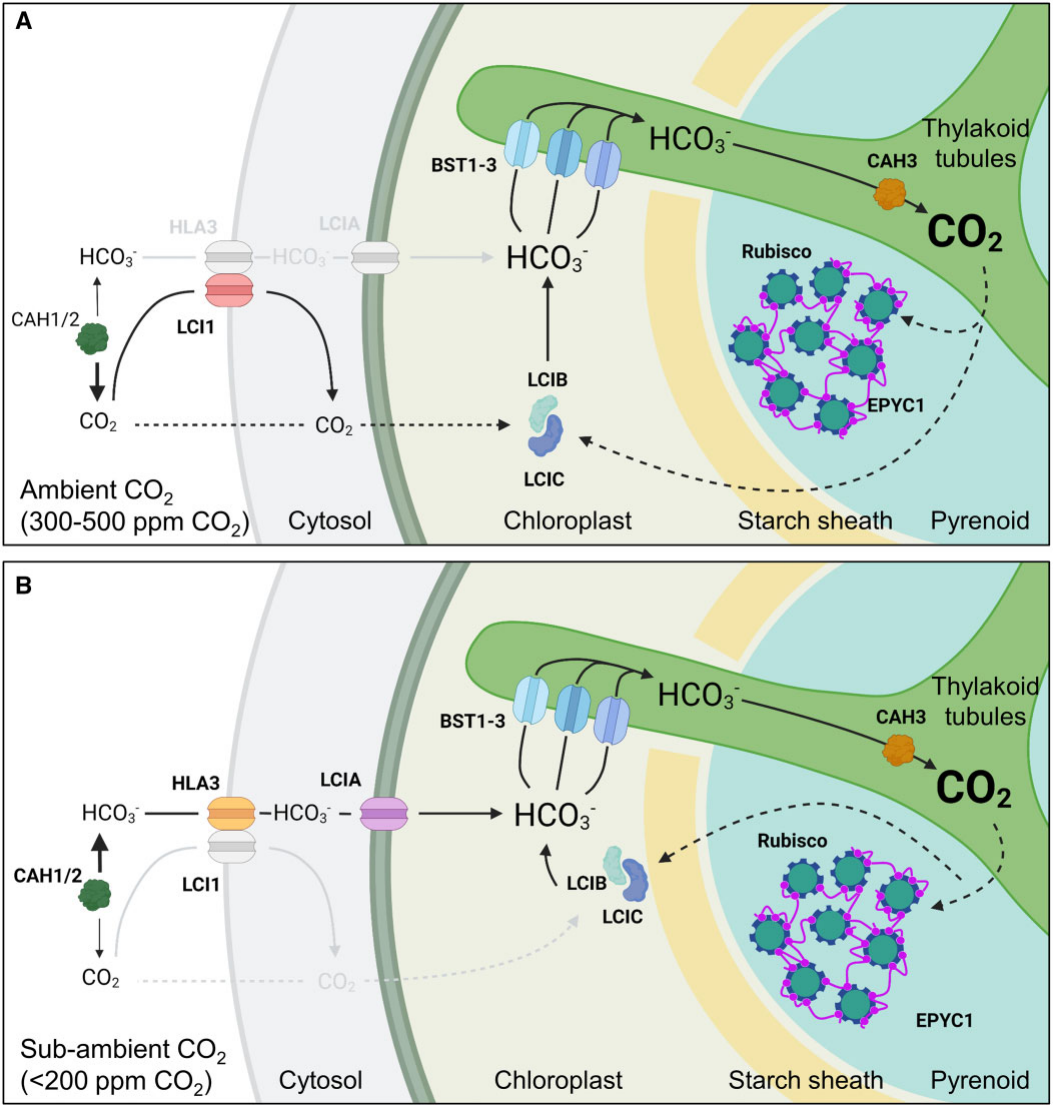
Overview of inorganic carbon uptake in the Chlamydomonas CCM. A, At ambient levels of CO_2_ (300–500 ppm, 0.03–0.05%, 10–18 µM CO_2_), extracellular inorganic carbon (Ci, i.e. CO_2_ and hydrated Ci, such as ${\text{HCO}}_{3}^{-}$) uptake is thought to be driven by drawdown of CO_2_ into the chloroplast through rapid conversion of CO_2_ to bicarbonate (${\text{HCO}}_{3}^{-}$) by LCIB, which is dispersed throughout the stroma in a complex with LCIC. ${\text{HCO}}_{3}^{-}$ is then transported into the lumen of thylakoid tubules traversing the pyrenoid by bestrophin-like channels (BST1-3) on the pyrenoid periphery. Carbonic anhydrase 3 (CAH3) located in the lumen within the pyrenoid converts ${\text{HCO}}_{3}^{-}$ to CO_2_, which diffuses into the surrounding Rubisco-EPYC1 matrix. CO_2_ not assimilated by Rubisco is converted back to ${\text{HCO}}_{3}^{-}$ by LCIB. Periplasmic carbonic anhydrases 1 and 2 (CAH1/2) and the plasma membrane CO_2_ channel low CO_2_-inducible protein 1 (LCI1) assist with inward CO_2_ diffusion (Fujiwara et al., 1990). Font sizes for CO_2_ and ${\text{HCO}}_{3}^{-}$ represent their relative concentration. B, At sub-ambient CO_2_ levels (<200 ppm, <0.03%, <7 µM CO_2_), the CCM transitions to an active ${\text{HCO}}_{3}^{-}$ uptake system that relies on the ${\text{HCO}}_{3}^{-}$ channels HLA3 protein and LCIA at the plasma membrane and chloroplast envelope, respectively. The LCIB/C complex relocalizes the pyrenoid periphery, and may interact with BST1-3 to rapidly recapture leaked CO_2_ as ${\text{HCO}}_{3}^{-}$ for re-uptake into the thylakoid lumen.

To overcome the limitations of Rubisco, numerous eukaryotic photosynthetic clades have evolved biophysical CCMs based on a pyrenoid micro-compartment, which actively work against a thermodynamic gradient to elevate the concentration of CO_2_ around Rubisco (Figure 1). Pyrenoid-based CCMs are highly abundant and estimated to be responsible for ca. 30% of global carbon capture (Barrett et al., 2021). Thus, they represent a promising diversity of systems for engineering yield improvements in C3 plants. Pyrenoids were among the first subcellular micro-compartments characterized by microscopy over 200 years ago (Vaucher, 1803). However, it was not until the early 1980s that the functional association of the pyrenoid with active Ci accumulation became apparent (Badger et al., 1977, 1978, 1980; Beardall et al., 1982). Badger et al. (1980) estimated that the algal CCM is capable of elevating Ci levels in the chloroplast stroma by 40-fold compared with ambient air. This is similar in range to that achieved by C4 plants. However, species with pyrenoid-based CCMs achieve this in a single cell as opposed to the two-cell Kranz-type anatomy typically required for the C4 pathway.

Pyrenoids consist primarily of Rubisco that has been aggregated into a liquid-like phase separated coacervate, or condensate (Freeman Rosenzweig et al., 2017). They range in size depending on species (1–2 μm in diameter), and shrink or expand dynamically in response to CO_2_ and light availability. Pyrenoids can be surrounded by a sheath of starch and are typically traversed by membranes that are continuous with the thylakoid network (Meyer et al., 2017).

The thylakoidal traversions are considered the primary source of Ci delivery, where ${\text{HCO}}_{3}^{-}$ is converted to CO_2_ in the acidic lumen to provide the surrounding Rubisco condensate with high levels of CO_2_ substrate. Pyrenoids are thought to have evolved several times, which may explain the wide diversity in structural characteristics across different lineages (Villarreal and Renner, 2012; Bordenave et al., 2021). These include differences in the organization of the traversing thylakoids, with some species showing complete absence of thylakoidal traversions, presence/absence of the starch sheath, and variations in starch plate shape and number when the starch sheath is present (Barrett et al., 2021). Nevertheless, several lines of evidence suggest that all pyrenoids are formed by liquid–liquid phase separation (LLPS). The shape of the pyrenoid matrix is always round or elliptical, which is characteristic of LLPS condensates. Furthermore, the available observational examples of dissolution and de novo assembly of pyrenoids consistent with Ostwald ripening (i.e. the thermodynamically favored growth of larger condensates at the expense of smaller condensates) support LLPS in a variety of different species (Osafune et al., 1990; Lin and Carpenter, 1997; Nassoury et al., 2001; Freeman Rosenzweig et al., 2017).

**Figure 2:**
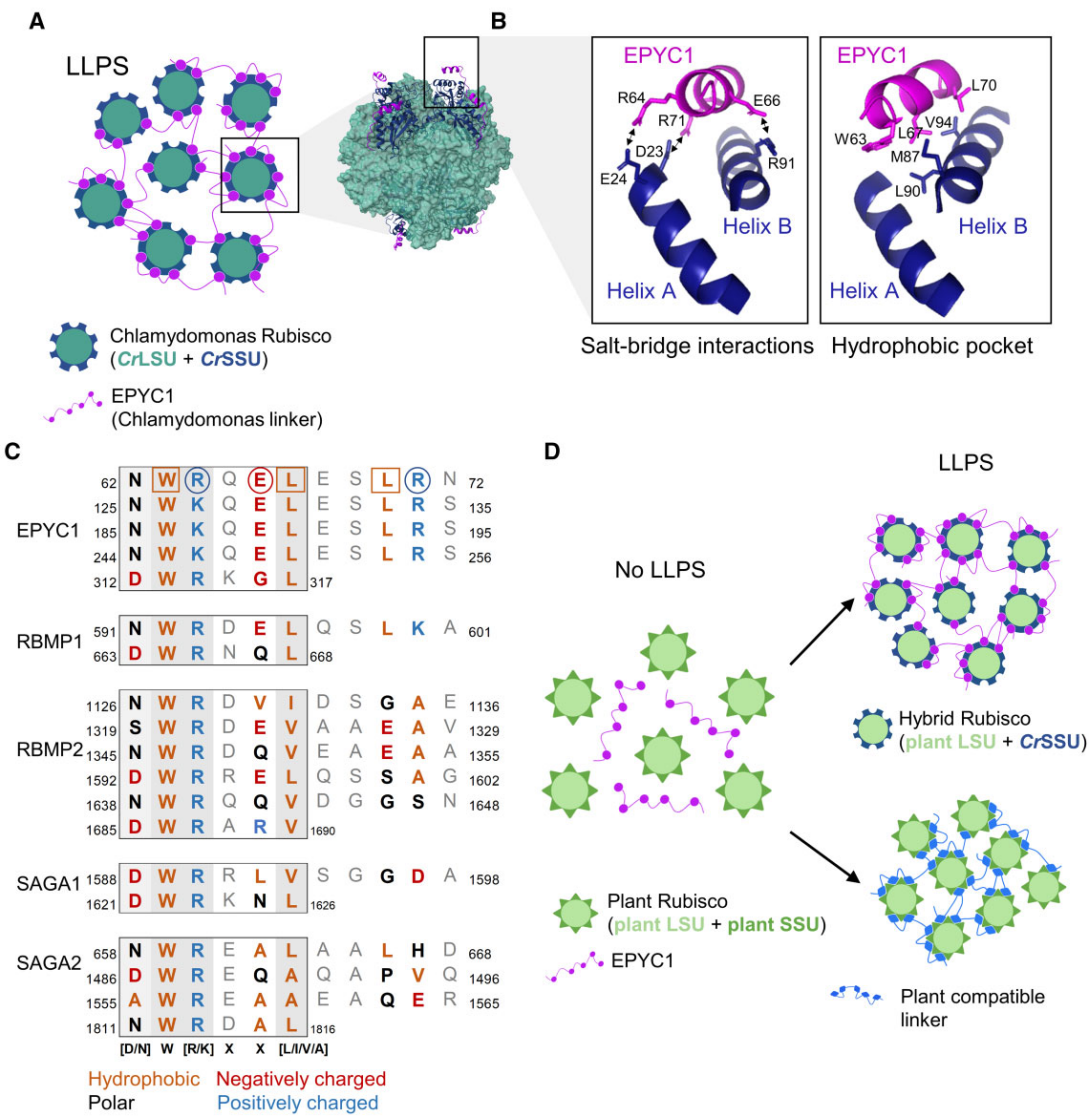
Rubisco condensation occurs through interactions between the Rubisco small subunit and the RBM. A, LLPS of Chlamydomonas Rubisco through multivalent interactions between the Rubisco small subunit (SSU) and the linker protein EPYC1. The model structure of EPYC1(49–72)-bound Rubisco was from Protein Data Bank entry 7JFO (figure made in ChimeraX). B, The Rubisco-binding motifs (RBMs) of EPYC1 and the two α-helices of the *Cr*SSU interact through key residues that facilitate salt-bridge interactions (left) and the formation of a hydrophobic pocket (right). C, The sequence diversity of RBMs within and between pyrenoid-localized proteins from Chlamydomonas. For RBMs from EPYC1 (top), the squared and circled amino acid residues form salt-bridge interactions and the hydrophobic pocket, respectively (as shown in B). The core motif is boxed, and residues that putatively interact with *Cr*SSU are bold and colored according to chemical properties. Shaded background indicates the level of conservation where darker shading indicates that the residue is more highly conserved. Numbers indicate the location of the motifs in the mature peptide. D, Strategies to achieve LLPS of plant Rubisco include the modification of plant Rubisco SSUs to generate a hybrid plant Rubisco compatible with EPYC1 (top right), or the generation of a linker protein with synthetic RBMs compatible with plant Rubisco SSUs (bottom right).

Apart from several seminal studies in diatoms and hornworts (Meyer et al., 2008; Hopkinson et al., 2011, 2014; Hopkinson, 2014; Young and Hopkinson, 2017; Li et al., 2020; Zhang et al., 2020b), the majority of molecular and physiological characterizations of pyrenoid-based CCMs, as well as our understanding of the regulation of pyrenoid formation, have been performed in Chlamydomonas (Figure 1). Induction of the CCM in Chlamydomonas is characterized by the maturation of a single pyrenoid that can sequester >90% of Rubisco pool around a knotted network of thylakoid tubules (Borkhsenious et al., 1998; Engel et al., 2015), which together are enclosed by a starch sheath consisting of several starch plates (Kuchitsu et al., 1988; Mackinder et al., 2017). Rubisco condensation is facilitated by the disordered linker protein Essential Pyrenoid Component 1 (EPYC1) (Mackinder et al., 2016). EPYC1 appears to interact exclusively with the small subunit of Rubisco (SSU) via Rubisco binding motifs (RBMs) that are common to several other pyrenoid components in Chlamydomonas (Figure 2, A and B; Meyer et al., 2012, 2020; Mackinder et al., 2016; Atkinson et al. 2019; He et al., 2020).

The structure of the binding site for EPYC1 and the Chlamydomonas SSU (*Cr*SSU) has been well characterized (He et al., 2020), and bioinformatic analysis has indicated that proteins with similar structural properties to EPYC1 (i.e. repeats of disordered domains followed by shorter, less disordered domains) occur in a broad range of algae (Mackinder et al., 2016). However, these putative linker proteins share little to no sequence similarity with EPYC1 or its RBM sites, except in close relatives (e.g. *Volvox* sp.). Similarly, the amino acid sequences of SSUs are relatively diverse and cannot be used to predict the presence of pyrenoids in other algae (Goudet et al., 2020). Together, the sequence diversity of the putative linkers and SSUs indicates that the RBM sites of linker proteins in other species and the mechanism(s) of interaction with Rubisco may be different. Further experimental characterization of putative condensing linker proteins and their interactions with Rubisco in other species could help to progress our understanding of the diversity of protein–protein interactions that facilitate Rubisco condensation.

**Figure 3:**
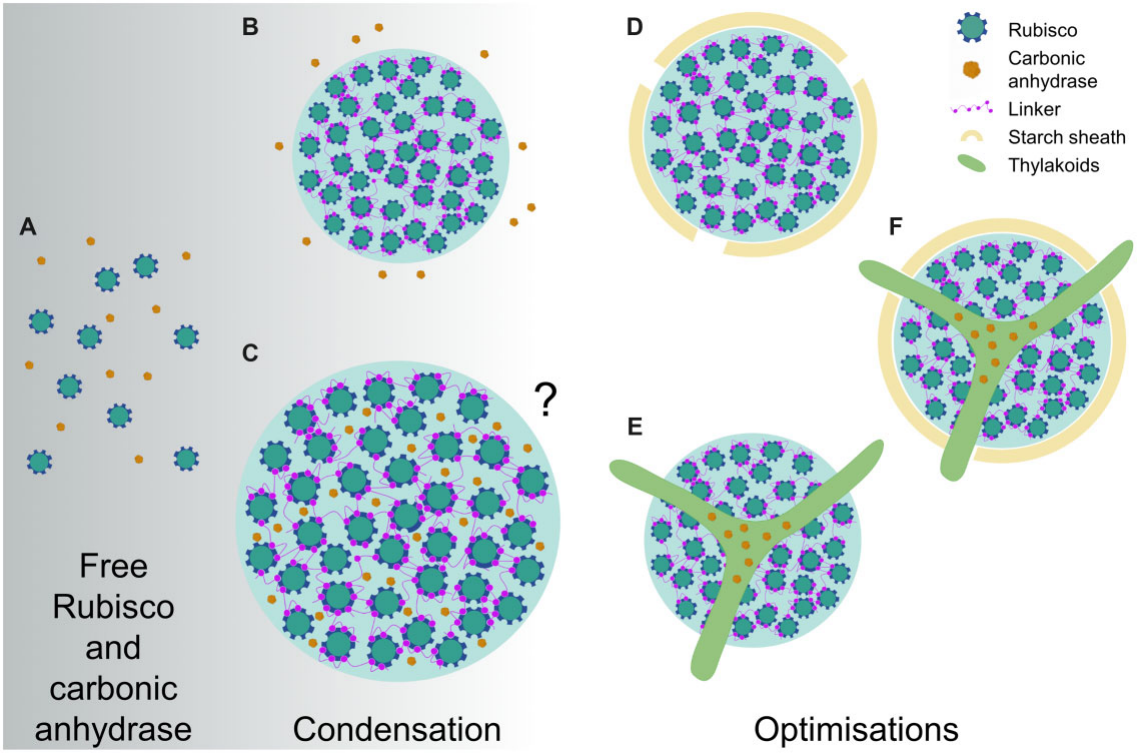
Hypothetical evolutionary pathways to pyrenoids from condensation. Model simulations propose that free Rubisco (A) and carbonic anhydrase (CA) could proceed to a phase separated condensate of Rubisco in the presence of a condensing protein factor/linker (e.g. EPYC1 in the Chlamydomonas pyrenoid, or CsoS2/CcmM in α/β-carboxysomes) with CA in close external proximity (B) or co-condensed inside the condensate (C) (Long et al., 2021). Due to the net proton release during Rubisco carboxylation and subsequent decrease in internal pH, co-condensation with CA would favor conversion of CO_2_ to ${\text{HCO}}_{3}^{-}$, and thus elevate CO_2_. Although the condensate could partially restrict outward diffusion, other models suggest that the condensate would have to be large (i.e. >3 µm in radius) or be surrounded by a diffusion barrier (Fei et al., 2022). Both models indicate that evolution of Rubisco condensation is feasible in the absence of additional Ci uptake components (e.g. LCIA and HLA3). Following condensation, pyrenoid evolution could proceed in several ways, including development of a starch sheath (D) to restrict diffusion of CO_2_ out of condensate, and/or a traversing thylakoid membrane that could allow regulatory re-localization of CA to within the condensate when required (E) (i.e. when the CCM is induced), and, in some cases, both combined (F). A comprehensive array of the diversity of pyrenoid architectures is illustrated in Barrett et al. (2021).

Modeling work has suggested that the evolution of Rubisco condensation may have preceded that of active Ci uptake systems, and that co-condensation of Rubisco and enzymes with carbonic anhydrase activity could facilitate an increased rate of Rubisco carboxylation in the condensate matrix (Long et al. 2021; Figure 3). The latter would be favored by the low pH maintained in the condensate due to the protons generated during carboxylation and the subsequent conversion of ${\text{HCO}}_{3}^{-}$ to CO_2_ by the carbonic anhydrase. As a counterpoint, more recent modeling efforts have indicated that such a condensate would need to be substantial in size (i.e. >3 µm in radius) to negate the rapid outward diffusion of protons and CO_2_ (Fei et al., 2022). Given that this radius is substantially larger than any known pyrenoid (e.g. Chlamydomonas pyrenoids seldom exceed 2 µm) and would require a large amount of Rubisco, this casts some doubt as to whether a condensate containing carbonic anhydrase could alone represent a primordial starting state for pyrenoid evolution. Nevertheless, the evolution of different pyrenoid architectures and accompanying CCM components may have proceeded from LLPS of Rubisco based on fitness gains (Figure 3). For example, the addition of a diffusion boundary around the pyrenoid matrix could reduce potential leakage of CO_2_ (Küken et al., 2018; Fei et al., 2022). Furthermore, the ability to regulate carbonic anhydrase levels in the matrix could increase capacity for environmental response and adaptation. In Chlamydomonas, this may have been achieved by the thylakoid tubules that transverse the pyrenoid, which are thought to facilitate the re-localization of the lumenal carbonic anhydrase 3 (CAH3) to the pyrenoid when the CCM is active (Karlsson et al., 1998; Blanco-Rivero et al., 2012; Sinetova et al., 2012). It remains unclear if the thylakoidal traversions observed in several algal and hornwort species are formed in response to the assembly of the pyrenoid. In Chlamydomonas, the tubule network remains intact in mutants that lack the capacity to form pyrenoids (Meyer et al., 2012), which suggests that the biogenesis of tubules is independent of the mechanisms that regulate Rubisco condensation.

## Chlamydomonas has two distinct CCM settings

**Figure 4:**
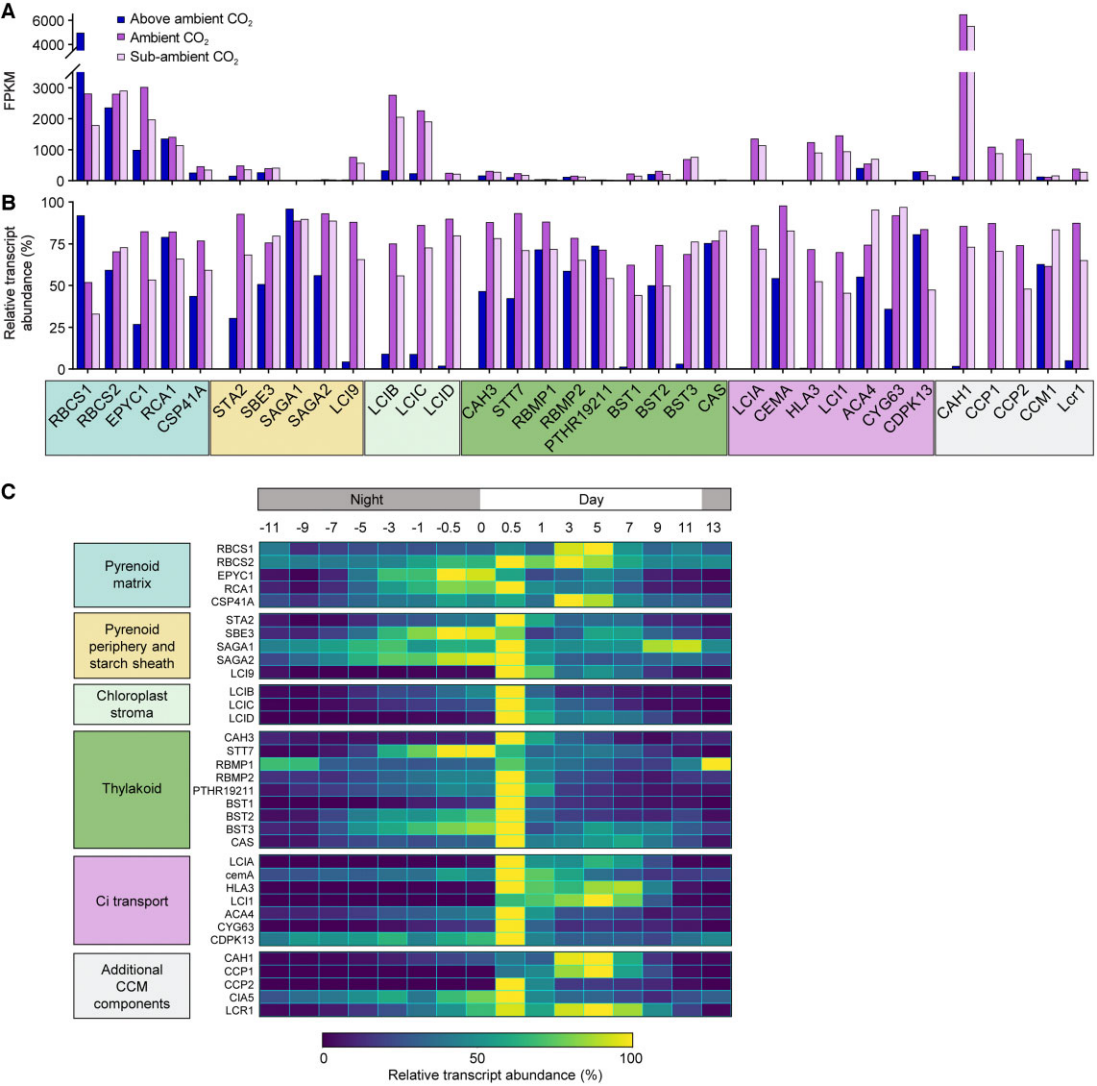
Expression of a selection of CCM genes under three different CO_2_ concentrations and over dark/light diel cycles. A, Absolute values for transcript levels in fragments per kilobase per million (FPKM) at above ambient CO_2_ (5%), ambient CO_2_ (0.03%–0.05%), and sub-ambient CO_2_ (0.01%–0.02%). Notably, the CCM genes highlighted here show a wide range of transcript abundances. B, Relative transcript abundance of each CCM gene. Data for A and B were derived from Fang et al. (2012). C, Relative transcript abundances in cultures grown at ambient CO_2_ (0.04%) over dark/light diel cycles (units refer to hours) (Strenkert et al., 2019). Genes are color coded according to localization and/or role in CCM function.

The Chlamydomonas CCM responds dynamically to Ci availability and light, and is also currently the only known CCM to transition between two pathways for Ci uptake depending on ambient Ci levels (Figure 1; Mackinder, 2017). Thus, biologists aiming to introduce the Chlamydomonas CCM into plants could potentially utilize components from one or both pathways. When Chlamydomonas cells are grown in above ambient CO_2_ concentrations (i.e. CO_2_-enriched air, 30,000–50,000 ppm CO_2_) or in the dark, the CCM is inactive. In these conditions, several CCM components show reduced transcriptional expression and the majority of the Rubisco pool is distributed throughout the chloroplast stroma (akin to C3 plants) (Borkhsenious et al., 1998). However, when CO_2_ concentrations are brought to ambient air levels in the light (i.e. 300–500 ppm or 10–18 µM CO_2_), Chlamydomonas cells undergo a major transcriptional and metabolic transition—the expression of many CCM-related genes is rapidly up-regulated (Figure 4, A and B), with a large proportion co-ordinated by the transcriptional regulator CIA5 (Ramazanov et al., 1994; Brueggeman et al., 2012; Fang et al., 2012; Strenkert et al., 2019). Under ambient CO_2_, the Chlamydomonas CCM is thought to function primarily in CO_2_, not ${\text{HCO}}_{3}^{-}$, uptake, which is dependent on the putative chloroplastic carbonic anhydrase LCIB (originally identified as low CO_2_-inducible B) (Figure 1A; Wang and Spalding, 2006; Jin et al., 2016). A further metabolic transition occurs when Ci levels are decreased to levels below ambient CO_2_ (i.e. <200 ppm CO_2_) (Spalding et al., 2002). In sub-ambient CO_2_, Chlamydomonas cells switch predominantly to an active ${\text{HCO}}_{3}^{-}$ uptake system mediated by the co-operative activities of LCIB and the putative Ci transporters high-light-activated 3 (HLA3) and low CO_2_-inducible A (LCIA) on the plasma membrane and chloroplast envelope, respectively (Figure 1B; Im and Grossman, 2002; Miura et al., 2004; Wang and Spalding, 2006; Duanmu et al., 2009a; Wang and Spalding; 2014a).

### The Chlamydomonas CCM is driven by LCIB at ambient CO_2_

LCIB homologs are found in a variety of species, including hornworts and diatoms, and have previously been characterized as a subgroup of the β-type carbonic anhydrase family (Jin et al., 2016, 2020; Li et al., 2020; Zhang et al., 2020a). Carbonic anhydrases catalyze the reversible interconversion of CO_2_ and ${\text{HCO}}_{3}^{-}$ (i.e. ${\text{HCO}}_{3}^{-}$ + H^+^ ↔ CO_2_ + H_2_O) in a pH-dependent manner; conversion to CO_2_ is favored below pH 6.4, while ${\text{HCO}}_{3}^{-}$ predominates at pH 6.4–10.3 (Moroney et al., 2011; DiMario et al., 2018; Momayyezi et al., 2020). In the light when the stroma is alkaline (i.e. pH 8) (Heldt et al., 1973), experimental evidence suggests that LCIB acts to draw Ci into the chloroplast by preferentially converting CO_2_ to ${\text{HCO}}_{3}^{-}$. As the permeability of the chloroplast envelope to ${\text{HCO}}_{3}^{-}$ is 10^4^ times lower than that for CO_2_ (Tolleter et al., 2017), ${\text{HCO}}_{3}^{-}$ is effectively trapped in the stroma. Thus, LCIB helps to maintain an inward CO_2_ diffusion gradient while increasing the overall stromal Ci concentration. In ambient CO_2_, LCIB is dispersed throughout the chloroplast stroma in a multimeric complex with low CO_2_-inducible C (LCIC) (Yamano et al., 2010, 2021). In support of its critical role, LCIB knockout mutants are unable to grow or assimilate CO_2_ in ambient CO_2_, whereas knockout mutations in LCIC appear to have no identifiable impact on growth or photosynthesis (Spalding, unpublished). The plasma membrane protein LCI1 also contributes to CO_2_ uptake in ambient CO_2_ (Ohnishi et al., 2010; Kono and Spalding, 2020), but is not essential for CCM function. Notably, recent determination of the structure of LCI1 has revealed that LCI1 forms a homotrimer consisting of four transmembrane domains that may function together as a gated CO_2_ channel (Kono et al., 2020).

In the chloroplast stroma, CO_2_ captured by LCIB as ${\text{HCO}}_{3}^{-}$ is thought to be channeled into the thylakoid lumen by the bestrophin-like transmembrane proteins, BST1, BST2, and BST3 (Mackinder et al., 2017; Mukherjee et al., 2019). All three BSTs are localized to the thylakoid membrane, including the tubules extending into the pyrenoid matrix, and are enriched around the periphery of the pyrenoid matrix. The relative contributions of each BST to ${\text{HCO}}_{3}^{-}$ uptake are still unclear. A knockout mutant of BST3 shows only a mild growth phenotype, which suggests some redundancy between the BSTs. However, RNAi triple-knockdown mutants for all three BSTs have a slow growth phenotype at ambient CO_2_ and a reduced Ci affinity compared with wild-type cells, which indicates that the BSTs co-operate to deliver ${\text{HCO}}_{3}^{-}$ to the lumen. Recent work by Burlacot et al. (2022) has revealed that BST-mediated ${\text{HCO}}_{3}^{-}$ uptake into the lumen is associated with consumption of the trans-thylakoid proton gradient that accrues when the photosynthetic electron transport chain is active in the light. The authors have suggested that the function of the BST channels may be energized by the proton motive force.

The lumenal α-CAH3 is then thought to convert ${\text{HCO}}_{3}^{-}$ accumulated in the acidic lumen (i.e. pH 6) into CO_2_, which would also be dependent on the available lumenal proton pool (Burlacot et al., 2022). Experimental evidence suggests that CAH3 is phosphorylated under ambient CO_2_, which results in a five- to six-fold increase in cellular carbonic anhydrase activity and relocation of CAH3 from the stromal thylakoids to the tubules traversing the pyrenoid (Blanco-Rivero et al., 2012; Sinetova et al., 2012). CAH3 appears to have optimal activity at a lower pH (i.e. 6.5) compared with other α-carbonic anhydrases and an acid disassociation constant (pKa) value of approximately 5.5 (Benlloch et al., 2015), which is consistent with adaptation to its presumed role in the lumen under light exposure. Thus, when the CCM is active, rapid conversion of ${\text{HCO}}_{3}^{-}$ to CO_2_ by CAH3 can maintain a ${\text{HCO}}_{3}^{-}$ gradient from the BST uptake sites into the pyrenoid tubules. Subsequent diffusion of CO_2_ out of the tubules generates a CO_2_-enriched environment for Rubisco in the pyrenoid matrix. CO_2_ that is not assimilated by Rubisco and diffuses out of the pyrenoid is presumably re-captured as ${\text{HCO}}_{3}^{-}$ by LCIB in the surrounding stroma.

Overall, when the LCIB-dependent CO_2_ uptake system is induced, the external Ci level required to achieve *V*_cmax_ in Chlamydomonas cells is substantially lower than that for those grown in CO_2_-enriched air (i.e. up to a 20-fold reduction in the Ci required for half maximal CO_2_ assimilation rates [*K*_0.5_]) (Coleman, 1984). In ambient CO_2_, cell doubling time remains similar to that in CO_2_-enriched air, while cell size is slightly decreased (Vance and Spalding, 2005). Notably, the transition point for CCM induction is relatively sharp—Vance and Spalding (2005) observed that photosynthetic rates just below 0.04% CO_2_ were markedly higher than for those just below 0.05% CO_2_.

### The Chlamydomonas CCM is complemented by active ${\text{HCO}}_{3}^{-}$ uptake at sub-ambient CO_2_

In sub-ambient CO_2_ (i.e. <0.03% CO_2_) LCIB can still contribute to CO_2_ uptake, but Chlamydomonas cells shift predominantly to an active ${\text{HCO}}_{3}^{-}$ uptake CCM system facilitated by HLA3 on the plasma membrane and LCIA on the chloroplast envelope (Figure 1B; Miura et al., 2004; Wang and Spalding, 2006; Duanmu et al., 2009b). The efficiency of the CCM increases further in sub-ambient CO_2_, as indicated by a further 13-fold reduction in *K*_0.5_ values compared with that at ambient CO_2_, although *V*_cmax_ values are reduced by 50% (Vance and Spalding, 2005). Chlamydomonas cultures grown in sub-ambient CO_2_ do grow more slowly than those at ambient CO_2_, and have smaller cells and less chlorophyll per cell.

Due to the complimentary roles of LCIB, LCIA, and HLA3 at sub-ambient CO_2_, single knockout mutations for these components are still able to grow (Wang and Spalding, 2006; Duanmu et al., 2009a; Yamano et al., 2015). For example, LCIB knockout mutants have wild-type-like CO_2_ assimilation rates and growth at sub-ambient CO_2_, in contrast to the lethal growth phenotype at ambient CO_2_. LCIA or HLA3 knockout mutants also have a normal growth phenotype under sub-ambient CO_2_ at neutral pH, but growth rates and Ci affinity are markedly reduced at high pH (i.e. pH 8.4–9) where ${\text{HCO}}_{3}^{-}$ is the predominant form of Ci available. A double LCIA/HLA3 knockout mutant shows a further decrease in growth compared with single mutants at high pH (Yamano et al., 2015). Together these results support the critical roles of LCIA and HLA3 specifically in ${\text{HCO}}_{3}^{-}$ uptake. Notably, double mutants of LCIB and LCIA are unable to survive in ambient or sub-ambient CO_2_ regardless of pH (Wang and Spalding, 2014a), demonstrating that disruption of both LCIB-dependent CO_2_ uptake and HLA3/LCIA-mediated ${\text{HCO}}_{3}^{-}$ uptake abolishes the CCM.

## CCM regulation in Chlamydomonas

Although several of the key components of the Chlamydomonas CCM are now characterized, how Chlamydomonas cells sense different external Ci levels is still poorly understood. Initiation of the CCM during a shift from above ambient CO_2_ to ambient CO_2_ is characterized by global changes in transcription (Brueggeman et al., 2012; Fang et al., 2012). In contrast, the expression levels of CCM components at sub-ambient CO_2_ are generally similar to those at ambient CO_2_, which indicates that the change in the operation of the CCM at sub-ambient CO_2_ is driven more by post-translational regulation (Figure 4, A and B). For example, HLA3/LCIA-mediated ${\text{HCO}}_{3}^{-}$ uptake appears to be rapidly inhibited during the transition from sub-ambient to ambient CO_2_, despite relatively small changes in transcript abundances (Wang and Spalding, 2014a). Recent work has also demonstrated that LCI1 is not important for CCM function in sub-ambient CO_2_ (Figure 1B; Kono and Spalding, 2020; Kono et al., 2020), even though transcription remains relatively high compared with that in above ambient CO_2_ (Figure 4A). Similarly, LCIB is relocalized from the stroma in ambient CO_2_ to a tight ring-like structure around the pyrenoid in sub-ambient CO_2_, while LCIB gene expression remains comparable between these conditions (Wang and Spalding, 2014b; Toyokawa et al., 2020). A more recent diel analysis of the Chlamydomonas transcriptome under ambient CO_2_ has shown that many CCM components are maximally expressed at the beginning of the day, while several components, such as EPYC1, are upregulated several hours prior to dawn (Figure 4C; Strenkert et al., 2019). This is consistent with previous work showing that the CCM can be fully induced before dawn in advance of maximum gene expression, which suggests that other mechanisms, such as circadian control, activate the CCM in anticipation of the coming day (Mitchell et al., 2014).

Post-translational modification and Ca^2+^ signaling are both implicated in regulating the transition between Ci uptake pathways at ambient or sub-ambient CO_2_, although current knowledge is somewhat fragmented. The migration of LCIB to the pyrenoid periphery is dependent on light and LCIC, and may be regulated by phosphorylation of itself, and/or LCIC within the LCIB/C complex (Jin et al., 2016; Yamano et al., 2021). Recent work has also highlighted the importance of the starch sheath in facilitating the migration of LCIB and CCM function (Toyokawa et al., 2020). LCI1 and HLA3 appear to form a multimeric complex on the plasma membrane with the putative calcium (Ca^2+^)-dependent ATPase transporter ACA4 (Mackinder et al., 2017). A Ca^2+^-binding, thylakoid membrane protein CAS has been implicated in a Ca^2+^-dependent retrograde signaling system that maintains and co-ordinates the expression of several nuclear-encoded CCM genes under limiting Ci, including LCIA and HLA3 (Wang et al., 2016). In above ambient CO_2_ or in the dark, CAS is dispersed across the thylakoid, but then moves to the thylakoid tubules within the pyrenoid under ambient and sub-ambient CO_2_ in the light (Yamano et al., 2018). Thus, expression of LCIA and HLA3 and other CCM components may be induced under ambient CO_2_ by CIA5 (Figure 4A), but induction of HLA3/LCIA-mediated ${\text{HCO}}_{3}^{-}$ uptake under sub-ambient CO_2_ appears to require a Ca^2+^ signal originating from within the pyrenoid. The transient receptor potential (TRP) Ca^2+^-channel TRP2, putatively located in the chloroplast envelope, could then relay signals originating from CAS to the cytosol (Christensen et al., 2020).

Recent work has also highlighted that retrograde signaling from the photorespiratory pathway and other feedback pathways (e.g. light stress) may play substantial roles in regulating CCM activity and the transition between the different CCM settings (Santhanagopalan et al., 2021). For example, Neofotis et al. (2021) observed that pyrenoid and starch sheath formation could be induced during hyperoxia even at high CO_2_ levels (i.e. 95% O_2_, 5% CO_2_), which indicated that CCM induction may be regulated by the products of photorespiration. In contrast, Ruiz-Sola et al. (2022) maintain that the intracellular Ci level is still the main factor regulating CCM induction, as CCM gene expression can be induced under low CO_2_ conditions even in the dark (i.e. in the absence of photosynthesis or photorespiration). Nevertheless, several studies now show that CIA5 is required for regulating the expression of photoprotection-, photorespiration-, and CCM-related genes, which suggests that the former processes are carefully co-ordinated with the CCM (Santhanagopalan et al., 2021; Redekop et al., 2022). Efforts to optimize the engineering of the Chlamydomonas pyrenoid-based CCM into C3 land plants may require due consideration of these interactions (Figure 4).

## Toward building a pyrenoid-based CCM in plants

Several models have predicted that introducing a biophysical CCM into a C3 plant chloroplast could substantially improve CO_2_ assimilation rates and increase crop yields (Price et al., 2011; McGrath and Long, 2014; Yin and Struik, 2017). These models are based on a carboxysome-based CCM and to date have been used as a proxy for the beneficial impact of a pyrenoid-based CCM. Compared with the CCM in C4 plants, which requires an additional two ATP per CO_2_ fixed, the introduction of a carboxysome-based CCM is predicted to be more energetically efficient. For example, expressing cyanobacterial Na^+^-dependent ${\text{HCO}}_{3}^{-}$ transporters BicA and SbtA on the chloroplast envelope might require an additional 0.25 ATP and 0.5 ATP per ${\text{HCO}}_{3}^{-}$ transported, respectively. These costs would be marginal when offset against the cost of photorespiration in C3 plants, provided that ${\text{HCO}}_{3}^{-}$ transport rates are matched to light availability (McGrath and Long, 2014), while Ci concentrations would be elevated in the chloroplast and increase the carboxylation efficiency of Rubisco. A fully functional carboxysome-based CCM (i.e. the additional encapsulation of Rubisco in carboxysomes and the removal of carbonic anhydrase activity from chloroplast except inside the carboxysome) could enhance the maximum achievable photosynthetic rates of C3 plants by 60% and crop biomass gains by 36%–60% depending on sink demand.

Recently, a reaction–diffusion model specifically based on the Chlamydomonas chloroplast was developed that has estimated the required components and the energetic costs for a functional pyrenoid-based CCM (Fei et al., 2022). Three modules were proposed as critical for CCM functionality: (1) a chloroplastic Ci uptake strategy that utilizes either a stromal carbonic anhydrase to drive a diffusive influx of CO_2_ into ${\text{HCO}}_{3}^{-}$ (i.e. the LCIB-dependent pathway) or an active pump to import ${\text{HCO}}_{3}^{-}$ (i.e. HLA3/LCIA-mediated ${\text{HCO}}_{3}^{-}$ uptake); (2) transport of ${\text{HCO}}_{3}^{-}$ into the thylakoid lumen via channels on the thylakoid membrane (i.e. BSTs) and its diffusion down into the tubules within the pyrenoid matrix to be converted by a carbonic anhydrase (i.e. CAH3) back into CO_2_; and (3) a pyrenoid matrix of condensed Rubisco, which is surrounded by diffusion barriers that restrict CO_2_ leakage. CCM configurations that either lacked or disrupted one of these three modules had an increased cost in ATP per CO_2_ assimilated and/or a reduced capacity to concentrate Ci.

**Figure 5:**
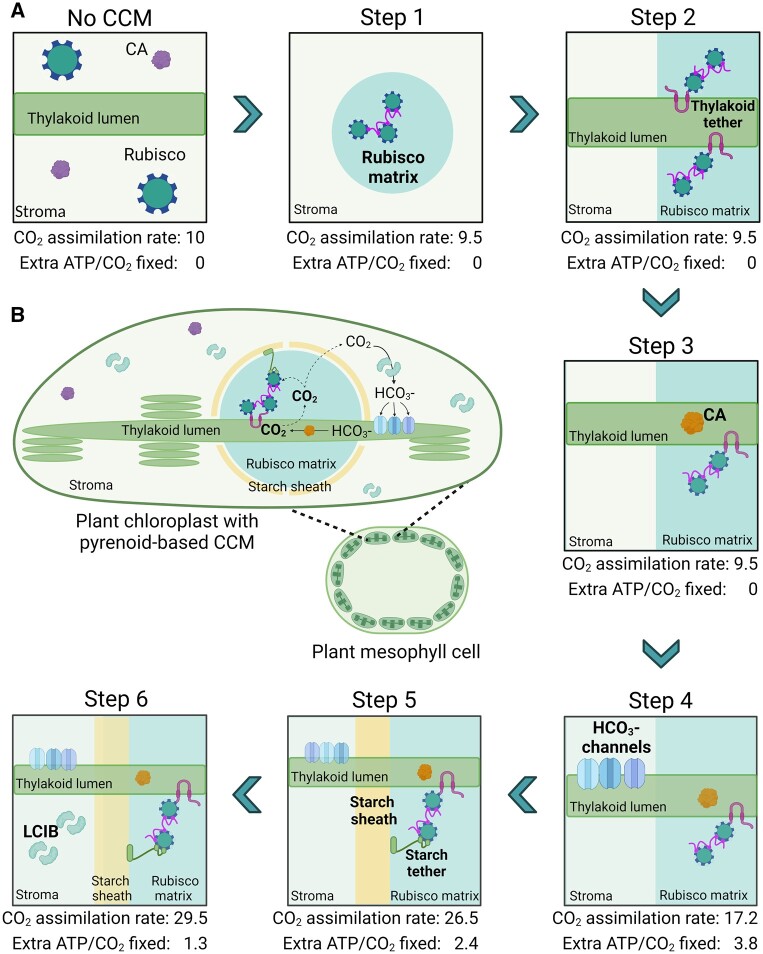
Pathway for engineering a pyrenoid-based CCM into C3 plant chloroplasts. A, A six-step strategy is shown for incorporating a minimal CCM into a C3 plant as based on the model proposed by Fei et al. (2022). For each step, the predicted leaf CO_2_ assimilation rate values are based on normalized CO_2_ fixation flux estimates, using an arbitrary starting value of 10 µmol CO_2_ m^−2^ s^−1^ for a wild-type C3 plant, and energy cost (i.e. additional ATP per CO_2_ fixed beyond that typically used in the CBB cycle). New additions in each subsequent step are highlighted in bold. A functional minimal CCM (i.e. step 6) is predicted to increase CO_2_ assimilation rates by three-fold with an energetic cost of 1.3 ATP per CO_2_ fixed. These values assume that native plant CA is excluded from the Rubisco matrix from step 1 and that CO_2_ assimilation rates are not limited by RuBP regeneration or triose phosphate utilization limitations. B, Schematic of a reconstituted minimal pyrenoid-based CCM in a C3 plant mesophyll cell chloroplast. Elements shown include Rubisco, native CA, LCIB, EPYC1, lumenal CA (e.g. CAH3) and starch or thylakoid tethers.

Based on an estimated CO_2_ concentration of ca. 10 µM in the cytosol of C3 mesophyll cells, Fei et al. (2022) demonstrated that a CO_2_ uptake strategy based on the “passive” LCIB-dependent pathway within the chloroplast would be sufficient and favorable for building a functional pyrenoid-based CCM in a C3 plant (Figure 5). This CCM could be driven solely by intercompartmental light-driven pH differences within the chloroplast, with the initial capture of CO_2_ as ${\text{HCO}}_{3}^{-}$ in the stroma by LCIB, or potentially native carbonic anhydrase activity. Importantly, the use of a passive Ci-uptake pathway could negate the requirement for active ${\text{HCO}}_{3}^{-}$ uptake at the chloroplast envelope and thus eliminate a key engineering challenge (Rottet et al., 2021). Successful introduction of the proposed pyrenoid-based CCM into a C3 plant is predicted to increase the Rubisco CO_2_ assimilation rate by up to three-fold at an estimated cost of 1.3 ATP per CO_2_ fixed. Below we will discuss a potential engineering path in terms of current progress and future challenges with a specific focus on implementing a passive Ci-uptake mechanism.

### Step 1: Rubisco condensation

A key requirement for introducing a pyrenoid-based CCM into plants is the capacity to aggregate the Rubisco pool into a phase separated condensate akin to the pyrenoid matrix. In Chlamydomonas, LLPS of the pyrenoid matrix is driven by weak multivalent interactions between α-helices A and B of the *Cr*SSU and five similar RBM sequences found on EPYC1 (Meyer et al., 2012; Mackinder et al., 2016; Atkinson et al., 2019; He et al., 2020). He et al. (2020) determined that three positively charged residues in the RBM form salt–bridge interactions with one and two negative charged residues on α-helix B and A, respectively (Figure 2, A and B). These interactions are supported by a hydrophobic pocket formed from three additional hydrophobic residues on the RBM and on α-helix B. Mutation of the residues contributing to the salt–bridges or the hydrophobic pocket either abrogates or substantially disrupts binding affinity. The overall binding affinity of EPYC1 for the *Cr*SSU may also be regulated by phosphorylation (Turkina et al., 2006; Wang et al., 2014). However, successful phase separation of recombinant EPYC1 with purified Chlamydomonas Rubisco in vivo has suggested that phosphorylation of EPYC1, or other post translation modifications, may be involved in reducing binding affinity to aid dissolution of the matrix rather than assembly.

The SSUs in plant species are structurally similar to those in Chlamydomonas, but differ in terms of sequence, surface charge, and hydrophobicity. For example, the α-helices in spinach SSUs are comparably more hydrophilic (Meyer et al., 2012), while the surface charges of rice SSUs are neutral or positively charged (Wunder et al., 2018). Thus, it is not surprising that EPYC1 has shown no evidence of interaction with plant SSUs, and efforts to phase separate EPYC1 with plant Rubiscos have not been successful (Wunder et al., 2018; Atkinson et al., 2019). Nevertheless, several studies have demonstrated that the large subunit (LSU) of Form 1B Rubiscos (found in plants, algae, and most cyanobacteria) is capable of assembling into functional Rubisco complexes with heterologous SSUs (Sharwood et al., 2008; Ishikawa et al., 2011; Orr et al., 2020). Although plant species typically have a family of nuclear-encoded SSU isoforms, recent work in tobacco and rice has shown that expression of a heterologous SSU can be combined with multiplex CRISPR approaches to replace dominant isoforms or the entire SSU family (Donovan et al., 2020; Matsumura et al., 2020). Atkinson et al. (2017) was able to complement an Arabidopsis mutant lacking two major SSU isoforms (i.e. 1A and 3B) with the *Cr*SSU gene *RbcS2* (Izumi et al., 2017). The resulting plants (S2_Cr_) contained a hybrid Rubisco pool consisting of approximately 50% *Cr*SSU and 50% *At*SSU. Although Rubisco in S2_Cr_ was characterized by small decrease in catalytic turnover rate (*k*_cat_^c^; Rubisco *V*_cmax_ per catalytic site) and specificity (*S*_C/O_), the values remained well within the range of Rubisco catalytic parameters observed for C3 plants (Orr et al., 2016). Furthermore, complementation with *RbcS2* restored Rubisco content and growth (in terms of rosette area) in S2_Cr_ to ca.70% of wild-type plants.

Subsequent introduction of EPYC1 into S2_Cr_ resulted in the formation of a condensate in the chloroplast consisting of hybrid Rubisco (i.e. *Cr*SSU:*At*LSU) that had similar LLPS properties to that of the Rubisco matrix in the Chlamydomonas pyrenoid (Atkinson et al., 2020). Thus, expression of EPYC1 and *Cr*SSU, with a reduction in native SSU levels, was sufficient to condense Rubisco into a single “proto-pyrenoid” in C3 plant chloroplasts. Furthermore, condensation of the hybrid Rubisco pool did not negatively affect plant growth, which is consistent with the predictions of the reaction–diffusion model (Figure 5A, step 1; Fei et al., 2022). Arabidopsis plants complemented with a modified, native SSU carrying α-helices A and B of *Cr*SSU were also able to form a condensate in the presence of EPYC1. The catalytic properties of Rubisco in the latter background were indistinguishable from wild-type plants, which suggests that native SSU families could be modified rather than replaced to generate “proto-pyrenoids” in other plant species with no negative impact on Rubisco performance.

As an alternative to engineering the SSU family, the RBM sites on EPYC1 could potentially be modified to interact with plant SSUs. The Chlamydomonas RBM has been found in several other pyrenoid-localized proteins and is quite variable in sequence [D/N]W[R/K]XX[L/I/V/A] (Figure 2C), yet still interacts with Rubisco (Itakura et al., 2019; Meyer et al., 2020). Furthermore, the RBM sequence can vary considerably within the same protein. Alignment of other RBMs with those from EPYC1 indicates that the residues critical for *Cr*SSU interaction, such as R71, can be different and have different properties (e.g. charge or hydrophobicity). This suggests that there may be alternative interaction mechanisms for different RBMs with Rubisco compared with those identified for EPYC1 RBMs and *Cr*SSU (He et al., 2020). If it is possible for *Cr*SSU to interact with a range of different RBMs, it may be feasible to design a synthetic linker with RBMs more suited to interaction with the α-helices of plant SSUs (Figure 2D).

### Step 2: Thylakoid association with the condensate

In Chlamydomonas, CO_2_ is delivered to the Rubisco condensate by diffusional release from the thylakoid tubule network that traverses through the pyrenoid (Engel et al., 2015). The model predicts that a complex network is not necessary for an efficient CCM, provided that a region of thylakoid membrane is in close proximity to pyrenoid matrix. These predictions are supported by the diversity of thylakoid architectures observed in other pyrenoid-based CCMs, and species such as diatom *Phaeodactylum tricornutum* and green alga *Chlorella vulgaris* where only a single thylakoid membrane traverses the matrix (Barrett et al., 2021). Thus, for plant engineering a key requirement is to bring a portion of the thylakoid membrane sufficiently close to the Rubisco condensate (Figure 5A, step 2).

Meyer et al. (2020) have proposed that the thylakoid–matrix interface in Chlamydomonas is formed by a series of “tethering” proteins that contain transmembrane domains to anchor to the thylakoid membrane and interact with the Rubisco matrix via RBMs. Rubisco Binding Membrane Proteins 1 and 2 (RBMP1 and RMBP2) are two putative candidates that appear to localize to the thylakoid tubule network within the pyrenoid matrix (Meyer et al., 2020). RBMP1 (71 kDa) is a BST-like channel homologous to BST1-3, but with an extended disordered C-terminal region that contains two RBMs. RBMP2 is considerably larger (165 kDa) and contains a long stromal C-terminal region with six RBMs. Although Meyer et al. (2020) have proposed that RBPM1 and/or RBMP2 could be involved in seeding matrix formation around the thylakoid tubules, it is also feasible that RBPM1 and/or RBMP2 are recruited to the pyrenoid matrix for a non-structural function. Further work is required to clarify which process might be occurring, and if additional components are involved in tethering. Nevertheless, tethering a Rubisco condensate to the thylakoid membrane in plants could be achieved with a combination of RBPM1, RMBP2, and/or synthetic protein tethers engineered to carry RBMs. For example, fusing three copies of the RBM to the stromal protein Ferredoxin-1 was sufficient for re-localization to the pyrenoid matrix in Chlamydomonas (Meyer et al., 2020). A synthetic tether for plants could be designed based on a native thylakoid transmembrane protein, such as the thylakoid protein kinase Stt7 (Lemeille et al., 2009).

### Step 3: Carbonic anhydrase in the thylakoid lumen

In Chlamydomonas, carbonic anhydrase activity within the thylakoid lumen traversing the pyrenoid (i.e. CAH3) is critical for CO_2_ delivery to the Rubisco matrix (Figure 1; Karlsson et al., 1998). Fei et al. (2022) observed that both localization of CAH3 within or close to the matrix and sufficient activity (i.e. >10^4^ s^−1^) were important to optimize the efficacy and energy efficiency of the CCM. These predictions correspond with the apparent enrichment of CAH3 within the pyrenoid when the CCM is induced and the optimal activity of CAH3 under low pH (i.e. when the acidity of the lumen increases in the light) (Blanco-Rivero et al., 2012; Sinetova et al., 2012; Benlloch et al., 2015). The presence of a lumenal carbonic anhydrase has been suggested in plants, specifically α-CA4 in Arabidopsis (Ignatova et al., 2019). However, evidence for localization of α-CA4 in the lumen is limited to a single proteomic analysis (Friso et al., 2004), while transcript abundances based on over 3,000 RNAseq experiments indicate near-zero levels of expression for α-CA4 (Zhang et al., 2020c). Therefore, a functional pyrenoid-based CCM in plants will likely require introduction of a carbonic anhydrase, optimized for lumenal expression, which localizes in close vicinity to the Rubisco condensate (Figure 5A, step 3). Due to the expected low abundance of ${\text{HCO}}_{3}^{-}$ in the thylakoid lumen of plants, the model predicts no substantial growth impact following expression of a lumenal carbonic anhydrase.

Previous work in *N. benthamiana* has indicated that CAH3 is not efficiently targeted to the thylakoid lumen (Atkinson et al., 2016). One possible explanation is that the native chloroplast transit peptide (cTP) of CAH3 is not sufficiently compatible for lumenal import in plants. Thus, the mature peptide of CAH3 could be fused to a plant native cTP to enhance the efficiency of translocation. Analysis of the cTP sequence of CAH3 indicates that translocation proceeds by the Tat pathway (Karlsson et al., 1998). Thus, fusion to a cTP from a similarly imported lumenal protein in plants, such as the 23-kDa protein of the oxygen-evolving complex of photosystem II (Morgenfeld et al., 2014, 2020), might be favorable. One further challenge will be to localize CAH3 to the thylakoid membranes associated with the Rubisco condensate. The importance of post-translational modification (e.g. phosphorylation) on CAH3 localization still requires further investigation in Chlamydomonas. However, CAH3 could potentially be anchored to the Rubisco condensate through fusion to or interaction with the lumenal side of tethering proteins discussed in step 2.

### Step 4: Bicarbonate channel(s) in the thylakoid membrane

BST1, BST2, and BST3 are thought to facilitate the flux of stromal ${\text{HCO}}_{3}^{-}$ into the thylakoid lumen (Figure 1; Mukherjee et al., 2019). Functional expression of one or more of the BSTs in the plant thylakoid membrane will be crucial to ensure a supply of ${\text{HCO}}_{3}^{-}$ for CAH3 to convert into CO_2_ (Figure 5A, step 4). Based on the known structures of other BST-like proteins, BST1-3 may each form a tetrameric or pentameric complex (Bharill et al., 2014; Mukherjee et al., 2019). Such complexes may be homomeric or a heteromeric combination of BST1-3 monomers. Further characterization of the functional roles of the three BSTs in Chlamydomonas will help to understand their apparent redundancy, and inform plant engineering strategies as to which BST(s) are required to support a functional CCM.

A key step for plant engineering will be to determine whether BST1-3 can localize to the thylakoid membrane in plants and to test their functionality as ${\text{HCO}}_{3}^{-}$ channels. Arabidopsis has two BST-like proteins (VCCN1 and VCCN2) that localize to the stromal lamellae (Duan et al., 2016; Herdean et al., 2016). This suggests that BST1-3 could integrate appropriately into plant thylakoid membranes via the same pathway as VCCN1/2 without the need for modification. However, functional characterization of heterologously expressed ${\text{HCO}}_{3}^{-}$ channels in planta remains challenging (Rottet et al., 2021). Burlacot et al. (2022) has demonstrated that the impact of the BST1-3 channels on thylakoid lumen pH could provide a method for assessing the function of BSTs in plant thylakoid membranes. For example, co-expression of a functional BST with a lumenal carbonic anhydrase (e.g. CAH3) may lead to increased consumption of the lumenal proton pool that would result in changes in non-photochemical quenching and proton motive force across the thylakoid membrane, which are routinely measured fluorescence parameters (Herdean et al., 2016).

### Step 5: A starch sheath diffusion barrier

The model predicts that a barrier around the pyrenoid is a key requirement to avoid diffusion of CO_2_ away from the Rubisco condensate before assimilation can occur (Fei et al., 2022). Addition of a diffusion barrier, modeled either in the form of starch sheath or thylakoid stacks, substantially improves the efficiency of the CCM by reducing CO_2_ leakage. However, under air-level CO_2_ (i.e. 10 μM cytosolic), a starch sheath is predicted to maintain 33% more CO_2_ around Rubisco than thylakoid stacks (Figure 5A, step 5). A starch sheath may also restrict inward diffusion of O_2_ from the chloroplast stroma, and thus suppress the oxygenation reaction of Rubisco (Toyokawa et al., 2020; Neofotis et al., 2021). As it is unclear in plants if the thylakoids surrounding the condensate will be sufficient to provide a CO_2_ diffusion barrier, it may be necessary to generate a starch sheath in planta.

In Chlamydomonas, starch formation and localization are dependent on environmental conditions. Stromal starch granules are typically dispersed throughout the chloroplast when the CCM is not induced, similar to the distribution of starch granules observed in plant chloroplasts in “stromal pockets.” However, under ambient CO_2_, starch initially accumulates around the pyrenoid as rounded granules, which eventually form into elongated starch plates (Ramazanov et al., 1994). During the formation of the starch sheath, the total starch content of Chlamydomonas cells appears to remain constant (Izumo et al., 2011), which suggests that the formation of starch around the pyrenoid is concomitant with stromal starch degradation. In contrast, when mixotrophically grown Chlamydomonas cells are nitrogen starved, new stromal starch granules begin to form, followed by breakdown of the starch sheath (Findinier et al., 2019). These observations suggest that partitioning of carbon between stromal starch granules and the pyrenoid starch sheath is dynamic in response to the growth environment.

The current model proposed for the recruitment of starch around the pyrenoid is via tether proteins, as proposed for the thylakoid–matrix interface by Meyer et al. (2020). Specifically, the proteins StArch Granules Abnormal (SAGA) 1 and SAGA2 have two and four RBMs, respectively, and both have a starch binding domain (SBD) near the N-terminus. SAGA1 and SAGA2 share 30% identity and both are located at the interface between the Rubisco matrix and the starch sheath. SAGA1 appears to localize in puncta at the periphery of matrix, whereas SAGA2 is more homogeneously spread across the matrix surface (Itakura et al., 2019; Meyer et al., 2020). SAGA1 and SAGA2 are thought to play key roles in organizing the morphology of the starch sheath. To date, only the *saga1* mutant in Chlamydomonas has been described in detail, which has elongated and thinner pyrenoid starch granules, and multiple pyrenoids (Itakura et al., 2019). It remains unclear if SAGA1 and/or SAGA2 would be sufficient for producing a starch sheath around a Rubisco condensate in planta, or if additional proteins putatively involved in pyrenoid starch metabolism would be required, such as STA2 (starch synthase 2), SBE3 (starch branching enzyme 3), and LCI9 (Mackinder et al., 2017). Further work should also focus on understanding the potential metabolic impact of producing starch around the Rubisco condensate and how this might compete with native chloroplastic starch turnover in plant chloroplasts.

### Step 6: CO_2_-uptake by stromal carbonic anhydrase

Four β-like carbonic anhydrase proteins are expressed in the chloroplast stroma in Chlamydomonas (i.e. LCIB, LCIC, LCID, and LCIE). The expression of LCID and LCIE is very low relative to LCIB and LCIC (Figure 3A; Spalding, 2007), while LCIC does not appear critical for CCM function. LCIB is currently considered the principal source of carbonic anhydrase activity in the stroma that captures CO_2_ as ${\text{HCO}}_{3}^{-}$ and drives the passive Ci-uptake mechanism (Atkinson et al., 2016; Mackinder et al., 2017), despite the conspicuous absence of quantified carbonic anhydrase activity in vitro (Jin et al., 2016). Fei et al. (2022) have estimated that the required stromal carbonic anhydrase activity for a functional CCM in Chlamydomonas is 10^3^ s^−1^ or larger, with a diffusion barrier in place around the pyrenoid matrix.

A key requirement of the model is the absence of stromal carbonic anhydrase activity in the Rubisco matrix to avoid conversion of CO_2_ to ${\text{HCO}}_{3}^{-}$, which would deplete CO_2_ in the pyrenoid matrix assuming that the local pH is comparable to the stroma (i.e. alkaline). This could be achieved by a barrier (e.g. a starch sheath, as in step 5) and potentially by molecular exclusion due to the increased density of the condensed matrix. For example, the pyrenoid matrix in Chlamydomonas appears to exclude proteins larger than 78 kDa that lack an RBM (Mackinder et al., 2017). This observation is consistent with the absence of LCIB within the matrix, which has been shown to form a large oligomeric complex with LCIC in vivo (350 kDa) (Yamano et al., 2010), or when purified from *Escherichia coli* (∼390 kDa) (Jin et al., 2016).

Plants already have an abundance of carbonic anhydrase proteins that can account for up to 2% of total soluble leaf protein (Momayyezi et al., 2020). Furthermore, the majority of leaf carbonic anhydrase activity is located in the chloroplasts, with the most highly expressed carbonic anhydrase β-type CA1 (βCA1) located in the stroma (Mergner et al., 2020). Thus, the activity of βCA1 could be sufficient to fulfil the role of LCIB and, following on from step 5, to functionalize the algal-based CCM (Figure 5A, step 6). Furthermore, β carbonic anhydrases also typically form oligomer complexes (DiMario et al., 2017), which may prevent diffusion into the Rubisco condensate.

If native stromal carbonic anhydrases are not sufficient, or inappropriate (e.g. due to size), for building a functional CCM, they could be replaced by LCIB and/or LCIC. Both proteins are able to self-localize to the chloroplast in plant mesophyll cells (Atkinson et al., 2016). However, if LCIB also proves sub-optimal to support a functional CCM in plant chloroplasts (e.g. due to inadequate activity from a lack of post-translational phosphorylation), alternative LCIB-like proteins could be employed. For example, homologs from the diatom *P. tricornutum*, *Pt*LCIB3 and *Pt*LCIB4, appear to be constitutively active (Jin et al., 2016). *LCIB* gene homologs are also present in hornwort species with active algal-like CCMs (Li et al., 2020). Although they are yet to be characterized, a functional LCIB isoform from a more closely related, early land plant chloroplast (e.g. *Anthoceros* spp.) could be better suited for driving CO_2_ uptake in a pyrenoid-based CCM for a C3 plant.

## Challenges and future prospects

Much progress has been made in our ability to engineer photosynthesis within a relative short timeframe, with yield improvements demonstrated from simple single gene manipulations to more integrated synthetic biology engineering strategies. The complex task of building a functional biophysical CCM has moved several steps closer to reality, particularly now with a model-based roadmap to guide future engineering efforts, and recent successes in understanding and transferring features of the Chlamydomonas CCM into C3 plants. Much is still unclear concerning the regulation of the Chlamydomonas CCM and how its activity is co-ordinated with other metabolic processes (see Outstanding Questions). Furthermore, many CCM components may require post-translational regulation to fine-tune function and appropriate localization. Progress in these areas will be critical to optimize the compatibility of a pyrenoid-based CCM with native plant metabolism. However, growing research interest to expand our understanding of pyrenoid-based CCMs in other species should also increase the availability of known components to build synthetic CCMs and further enhance the prospect of exciting advances in the near future.

ADVANCES

- A model-guided engineering path has been developed for introducing a biophysical CCM into C3 chloroplasts that could increase leaf CO_2_ assimilation rates by three-fold with a negligible ATP cost.
- Molecular interactions that facilitate phase separation of the pyrenoid condensate in Chlamydomonas and reconstitution of a proto-pyrenoid in Arabidopsis have been characterized.
- A Rubisco-binding motif that can localize proteins to the pyrenoid matrix has been identified in Chlamydomonas.

OUTSTANDING QUESTIONS

- Can an interaction interface between native crop Rubiscos and an EPYC1-like linker protein be engineered?
- Is the native level of stromal carbonic anhydrase activity in plant chloroplasts sufficient to drive a pyrenoid-based CCM?
- How are thylakoid membranes that traverse pyrenoids formed and what is the function of the traversing thylakoids?
- What regulates the formation and turnover of starch around pyrenoids? Will a starch sheath interfere or integrate with plant starch metabolism?
- How is the lumenal carbonic anhydrase CAH3 re-localized to the pyrenoid thylakoid tubules in Chlamydomonas?
- How is a pyrenoid-based CCM energized? Will normal levels of ATP production and the proton motive force in C3 thylakoids be sufficient to power a CCM?
- What are the minimal components required for a functional pyrenoid-based CCM?

## Funding

A.J.M. acknowledges funding from the UKRI Biotechnology and Biological Sciences Research Council (grant number BB/S015531/1) and Leverhulme Trust (grant no. RPG-2017-402). L.A. was funded by the BBSRC East of Scotland Bioscience (EASTBIO) Doctoral Training Partnership program. C.F. was funded by the NSF through the Center for the Physics of Biological Function (PHY-1734030).

*Conflict of interest st* *atement*. The authors have no conflicts to declare.

## Notes

L.A., A.D.-R., and Y.M. contributed to the design, writing, and the generation of figures. K.R.P. and C.F. contributed to the acquisition and interpretation of data, generation of figures, and manuscript revision. A.J.M. supervised and contributed to writing, revision, and final approval.

The author responsible for distribution of materials integral to the findings presented in this article in accordance with the policy described in the Instructions for Authors (https://academic.oup.com/plphys/pages/general-instructions) is: Alistair J. McCormick (alistair.mccormick@ed.ac.uk).
